# Verbally Highlighting Extrinsic Causes of Novel Social Disparities Helps Children View Low-Status Groups as Structurally Disadvantaged Rather Than Personally Inferior

**DOI:** 10.3389/fpsyg.2021.716662

**Published:** 2021-10-13

**Authors:** Rebecca Peretz-Lange, Paul Muentener

**Affiliations:** ^1^Department of Psychology, SUNY Purchase College, Purchase, NY, United States; ^2^Department of Psychology, Tufts University, Medford, MA, United States

**Keywords:** social essentialism, attribution, explanation, verbal framing, structural reasoning

## Abstract

As part of their “essentialist” intuitions, young children tend to form *personal attributions* for observed intergroup differences – attributing them to groups’ intrinsic natures or inborn characteristics. Much research has linked this essentialist view of social groups with prejudiced attitudes. However, less research has explored children’s capacity to form *structural attributions* for observed intergroup differences – attributing them to groups’ extrinsic circumstances or access to opportunities – or how structural attributions relate to social attitudes. Structural attributions could enable children to view low-status groups as extrinsically disadvantaged rather than intrinsically inferior. We were interested in whether verbally highlighting the extrinsic causes of novel social status disparities could support young children in forming structural attributions for them, thereby mitigating the formation of prejudice toward novel low-status groups. To investigate, we introduced participants (*n*=106 5- and 6-year olds) to novel social status disparities that could be attributed to either intrinsic or extrinsic causes, and we framed the disparities in either intrinsic, neutral, or extrinsic terms. We then assessed children’s attributions for the disparities (through two measures: explanations and interventions) and their social attitudes toward the groups (through two measures: friendship preferences and prize allocations). Results indicated that participants tended to provide mostly personal attributions overall but that extrinsic framing led them to provide significantly more structural attributions. Extrinsic framing did not significantly impact social attitudes overall, but exploratory analyses revealed that it impacted participants’ friendship preferences in particular. Together, results suggest that verbally highlighting extrinsic causes can disrupt children’s intuitive tendency toward personal attributions, with promising implications for their views of low-status groups.

## Introduction

By the preschool years, children tend to hold an intuitive “essentialist” view of social groups, which includes attributing observed intergroup differences to group members’ *intrinsic* natures. This attributional reasoning may promote prejudice. In the present study, we draw from research showing that verbal framing can influence children’s causal attributions, and we investigate whether framing can be used to highlight *extrinsic* causes of intergroup differences, and in turn, to mitigate prejudice development.

### Personal Attributions and Prejudice Development

Young children tend to view social groups (e.g., women and Black people) through an “essentialist” lens: They intuitively view these groups as natural and assume that group members share an underlying “essence” that gives rise to their observable similarities (see Gelman, 2003 for a review). For example, when told a story about a baby girl raised exclusively by men, preschoolers report that the baby will nevertheless grow up to exhibit gender-stereotypical behaviors like playing with dolls (Taylor, 1996; Taylor et al., 2009), thus ignoring the role of the extrinsic environment in shaping behavior. Moreover, when asked to explain their reasoning, preschoolers reference girls’ blood or “kind of stuff inside” their bodies (Gelman and Wellman, 1991). Children thus tend to view intergroup differences as explained by people’s insides or biology, rather than their outsides or circumstances. Children apply these essentialist intuitions not only to gender but also to race (Hirschfeld, 2008; Mandalaywala et al., 2019), ethnicity (Diesendruck and Menahem, 2015; Segall et al., 2015), spoken language (Kinzler and Dautel, 2012), and religion (Heiphetz et al., 2017; Smyth et al., 2017).

A wide literature has linked social essentialism with prejudice (see Rhodes and Mandalaywala, 2017 for a review). For example, 3–10-year-old children’s essentialist beliefs about race predicted their use of negative racial stereotypes (see also, Levy and Dweck, 1999; Pauker et al., 2010; Mandalaywala and Rhodes, 2016). In an experimental study, Diesendruck and Menahem (2015) assigned Israeli 6-year olds to read stories that either did or did not emphasize essentialism of ethnic groups (Jews and Arabs), and they found that children who heard the essentialism-emphasizing story later drew outgroup members with less positive affect in a drawing task, indicating prejudice.

One mechanism through which essentialism may promote prejudice is by shaping children’s attributions for social status disparities they observe. Children are highly attuned to intergroup disparities in social status, such as disparities in social dominance, achievement, wealth, and social power (Nesdale and Flesser, 2001; Olson et al., 2012; Shutts et al., 2016; Enright et al., 2020; Mandalaywala et al., 2020; we return to the multifaceted nature of social status in the Discussion). They robustly hold attitudes favoring high- over low-status groups (Horwitz et al., 2014; Li et al., 2014; Shutts et al., 2016; Ahl and Dunham, 2019), even when these groups are novel (e.g., Blues and Yellows, Bigler et al., 2001). Guided by their essentialist intuitions, children may form *personal attributions* for status disparities, attributing them to groups’ intrinsic abilities or personal characteristics. In turn, children may conclude that high-status groups must be intrinsically superior to low-status groups and then form attitudes favoring the groups they view as superior. Indeed, researchers have long proposed that incorporating observed intergroup status disparities into one’s essentialist representations in this way may contribute to prejudice toward low-status groups (Allport et al., 1954; Rothbart and Taylor, 1992; Pratto et al., 1994).

Critically, if children’s *attributions* for status disparities underlie their status-based social attitudes, then manipulating their attributions may be a way to improve their social attitudes. We investigate this possibility in the present study by manipulating children’s attributions and assessing consequences on their social attitudes. In particular, we investigate whether the verbal framing of social status disparities can shift children’s attributions for them. We are motivated by the research showing that children form and revise their causal knowledge in social collaboration with parents and caregivers (see Legare et al., 2017 for a review), and that children can learn and generalize new types of explanations when provided with examples of them from an adult (Walker et al., 2015; Lombrozo et al., 2018). In particular, we draw from research demonstrating that verbally highlighting extrinsic causes can disrupt children’s default tendency to form personal attributions (Vasilyeva et al., 2018; Peretz-Lange and Muentener, 2019). For example, Peretz-Lange and Muentener (2019) verbally framed characters’ behaviors in either intrinsic, extrinsic, or neutral terms, and then assessed preschoolers’ attributions for the behaviors. They found that framing impacted attributions asymmetrically; children provided similarly high rates of personal attributions in the intrinsic and neutral framing conditions, in line with their default attributional tendencies, but provided significantly fewer attributions in the extrinsic framing condition. We use a similar manipulation in the present study. We expect that extrinsic framing will similarly mitigate children’s tendency toward personal attributions relative to intrinsic and neutral framing.

We are not the first to manipulate social essentialist reasoning and examine impacts on prejudice. However, prior work has typically compared an essentialist condition (i.e., one promoting essentialist views) with a non-essentialist condition (i.e., a control condition; e.g., Bastian and Haslam, 2006; Rhodes et al., 2012; Diesendruck and Menahem, 2015; Rhodes et al., 2018; Mandalaywala et al., 2019). Instead, the present study compares an essentialist condition, a non-essentialist control condition, *and an anti-essentialist* condition in which structural attributions are induced in place of personal attributions. In doing so, we investigate whether promoting an alternative causal attribution for observed disparities may be an effective way to actively reduce essentialist reasoning, going a step further than merely avoiding reinforcing essentialist views.

### Structural Attributions as an Alternative to Personal Attributions

Structural attributions involve viewing social phenomena as caused by *extrinsic* factors such as groups opportunities, circumstances, or treatment. We propose that children who form structural (rather than personal) attributions for social status disparities will conclude that low-status groups are merely extrinsically disadvantaged (rather than intrinsically inferior), supporting more egalitarian social attitudes. In other words, structural attributions might enable children to causally account for observed disparities while avoiding personal attributions and their pernicious consequences. Optimistically, emerging research supports the idea that even young children are capable of forming structural attributions (Hussak and Cimpian, 2015; Vasilyeva et al., 2018; Peretz-Lange and Muentener, 2019; Yang et al., 2021). The present study contributes to this emerging research area in a few ways:

First, the research on structural attributions often involves explicitly telling children about the cause of a given disparity (e.g., Hussak and Cimpian, 2015; Sutherland and Cimpian, 2019; Rizzo et al., 2020; Dunlea and Heiphetz, 2021). However, as recent reviews have pointed out (Elenbaas et al., 2020), children rarely receive explicit explanations for disparities; instead, they are often faced with causally-ambiguous disparities and left to their own devices to make sense of them. In the current study, children were not provided with explicit explanations for the presented disparities. Instead, disparities were designed to be equally attributable to either an intrinsic or an extrinsic cause, and the verbal framing varied between conditions to more subtly highlight either an intrinsic or an extrinsic factor or neither.

Second, most research on structural reasoning focuses on real-world groups (e.g., gender, see Vasilyeva et al., 2018; Rizzo et al., 2020; Amemiya et al., 2021; Yang et al., 2021) and real-world disparities (e.g., incarceration, Dunlea and Heiphetz, 2021; school achievement, Goudeau and Cimpian, 2021). The use of real-world groups has important benefits for external validity. However, this approach also has some limitations for basic research, as it does not indicate whether attributions drive the *initial formation* of social attitudes or merely reinforce preexisting social attitudes or preexisting essentialist beliefs. By using novel groups and novel structures, the present study circumvents any preexisting beliefs or attitudes that children may hold.

A final way that the present study builds on existing research is through its inclusion of social attitude measures. Research has demonstrated promising consequences of structural reasoning on a variety of outcomes, including children’s moral evaluations of disparities (Elenbaas et al., 2016), tendency to perpetuate disparities (Elenbaas et al., 2016; Rizzo et al., 2020), view of disparities as mutable (Vasilyeva et al., 2018; Yang et al., 2021), evaluations of nonconformity (Yang et al., 2021), and support for reducing structural obstacles (Elenbaas, 2019). In the present study, we examine impacts on social attitudes. Given the effects essentialist reasoning, and status information in general, on social attitudes, we were interested in whether structural attributions could mitigate these effects.

### The Present Study

In the present study, we introduced children to novel social status disparities. Specifically, these were social dominance disparities, featuring one group becoming dominant and another becoming subordinate in a novel game. We drew from past research using winning/losing to operationalize status in novel contexts (e.g., Thomas et al., 2016, 2018) and evidence that infants and non-human animals use winning/losing to track social dominance hierarchies (Thomsen, 2020), but we note the limitations of this approach (see section Discussion). Critically, we designed each disparity to be explained equally well by either an intrinsic or an extrinsic cause. Between-subjects, we verbally framed the disparities to highlight either an intrinsic cause, an extrinsic cause, or neither. Then, we assessed children’s causal attributions for the status disparity (through two measures: explanations and interventions), and finally, their social attitudes toward the dominant versus subordinate group (though two measures: friendship preferences and prize allocations). This design allowed us to explore how verbal framing impacts personal vs. structural attributions, and in turn, social attitudes.

**Figure 1:**
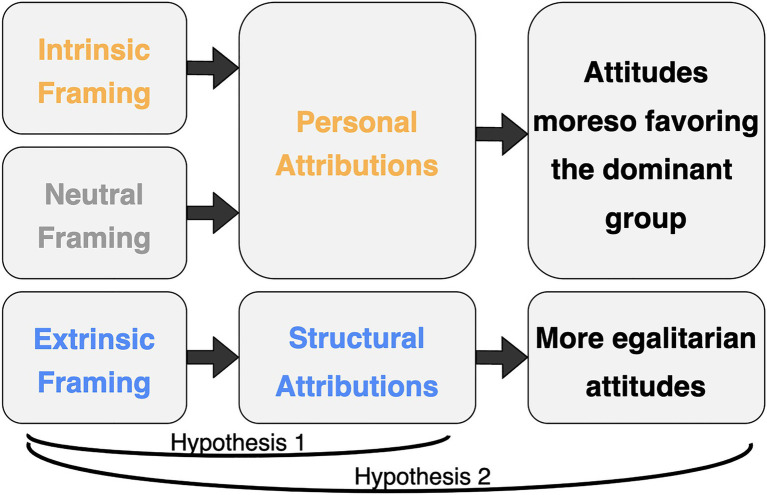
Schematic of hypotheses.

We had two primary hypotheses (Figure 1). First, we expected framing to impact children’s attributions. In particular, we expected children to provide mostly personal attributions following intrinsic and neutral framing, but relatively more structural attributions following extrinsic framing. Second, we expected framing to also asymmetrically impact children’s attitudes, with intrinsic and neutral framing leading children to favor the dominant groups moreso than extrinsic framing. We made no *a priori* predictions about differences in our two measures of attributions (explanations and interventions, drawn from prior work using both measures, e.g., Harris et al., 1996; Nyhout and Ganea, 2019) or our two measures of social attitudes (friendship preferences and prize allocations drawn from prior work finding internal reliability on similar measures, e.g., Hussak and Cimpian, 2015).

As secondary aims, we were also interested in participants’ ability to generalize the verbal framing they received to a new unframed scenario and in age-related changes in participants’ attributions. We predicted that participants would successfully generalize the framing they received to a new scenario, as in prior work (Seiver et al., 2013; Peretz-Lange and Muentener, 2019). This would indicate that framing shapes participants’ conceptual understanding of disparities, rather than only their perceptual attention to different features of the visual scene, though we do not claim this indicates any longer-lasting impacts of framing outside of the context of the experiment. We also predicted that structural attributions would increase over development, as in prior work on structural attributions for gender differences (Vasilyeva et al., 2018; Amemiya et al., 2021; Yang et al., 2021).

We tested 5- and 6-year-old participants, reasoning that social essentialism and status-based social preferences are both in place by the preschool years (Rhodes and Mandalaywala, 2017; Enright et al., 2020), but that younger children might find the verbal and working memory demands of the tasks challenging. We also aimed to build upon related work on children in similar age ranges (e.g., Rhodes et al., 2018; Peretz-Lange and Muentener, 2019).

## Materials and Methods

### Participants

A total of 106 5- and 6-year-old children (mean age=5years 11months, 54% male and 46% female) participated in this study. Participants were recruited from local museums in the Boston area. Gender and birthdates were reported by participants’ parents and guardians, and birthdates were used to calculate participants’ exact ages at the time of the study. Although museum policy precluded collecting other demographic information from participants, demographic information about the museum visitor population suggest that our sample was likely largely White and from middle- to upper-class backgrounds. Oral consent was obtained from all participants, along with a written consent from their parents or guardians. This consenting procedure was approved by the Social, Behavioral, and Educational Institutional Review Board at Tufts University.

An *a priori* power analysis revealed that a sample of 105 participants would be sufficient to detect a medium-small effect at a power level of at least 80%. Participants were randomly assigned to either the intrinsic (*n*=35), neutral (*n*=36), or extrinsic (*n*=35) framing condition. This sample size follows prior work manipulating young children’s structural reasoning, which has employed sample sizes of 20 (Hussak and Cimpian, 2015; Vasilyeva et al., 2018) and 24 (Rizzo et al., 2020) participants per condition within the age range analyzed. We used a slightly larger sample size, powered to detect a medium-small effect, as we were interested in downstream effects of our manipulation on children’s social attitudes.

### Materials

**Figure 2:**
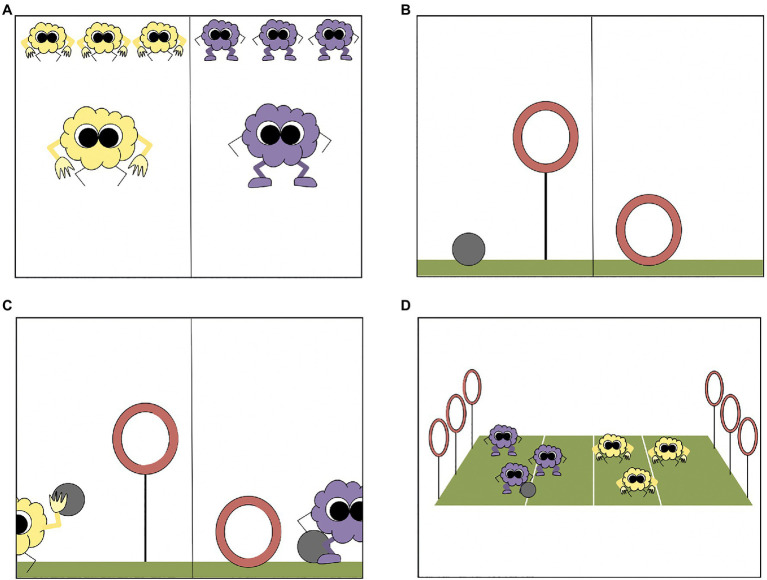
Trial 1 slides. Slides depicted **(A)** an intrinsic difference between novel groups, **(B)** an extrinsic difference between novel structures, **(C)** a demonstration of both differences as they related to status, and **(D)** the process of status differentiation.

The stimuli for this study consisted of a series of animated slides created on Microsoft PowerPoint. Three different sets of four animated slides comprised the three trials. Slides were presented on a laptop computer in a fixed order and were identical across conditions. The set of slides used in Trial 1 are shown in Figure 2, but Trials 2 and 3 involved different sets of slides featuring different characters and structures.

### Procedure

All participants completed three trials, each consisting of a familiarization phase, a status differentiation phase, and a test phase described below. Note that the experimental manipulation of verbal framing occurred only during the status differentiation phase; the verbal narration accompanying the procedure was otherwise identical across conditions.

The first two trials included verbal framing, but the third trial did not, and was considered as a generalization trial. This third trial involved only a familiarization phase and test phase, but no status differentiation phase, and therefore no experimentally manipulated framing.

#### Familiarization Phase

First (Figure 2A), participants were told about two novel groups of characters, referred to by their colors using generic language (e.g., “In this game, there are two kinds, the Yellows and the Purples”). Next, participants were told about an intrinsic difference between these groups as it related to a difference in ability (e.g., “Guess what? Yellows have really strong arms, so they are really good at throwing, and Purples have really strong legs so they are really good at kicking”). As an attention check, participants were then asked to recall each group’s ability (e.g., “So, who is good at kicking? … at throwing?”)

Next, (Figure 2B), participants were told that the groups were going to play a game against each other, and that two novel structures were involved in the game (e.g., “There are two ways to get points in this game, high-up hoops and low-down hoops”). Participants were told about an extrinsic difference between these structures as it related to a difference in the ability required to earn points (e.g., “Guess what? High-up hoops are for throwing a ball into, and low-down hoops are for kicking a ball into”). As an attention check, participants were then asked to recall each structure’s required ability (e.g., “So, which hoop is for kicking? … for throwing?”).

Finally, (Figure 2C), both sets of differences were demonstrated (e.g., a Yellow would throw a ball into a high-up hoop, and a Purple would kick a ball into a low-down hoop).

#### Status Differentiation Phase

Participants were told, “Ready to watch them play and see who wins?” and shown the process of gameplay (Figure 2D). In the game, one extrinsic structure of the game was not used (e.g., no low-down hoops) and the corresponding intrinsic ability was not useful (e.g., no use for kicking). One group made three successful attempts (earning three points) and the other group made three unsuccessful attempts (earning zero points), resulting in a decisive victory. Critically, this victory could always be rationally attributed to an intrinsic difference (characters’ bodies or abilities) or an extrinsic difference (structures’ forms or required abilities). Both causes were always equally accurate explanations.

The experimental manipulation involved the verbal framing narrating the gameplay, which varied between conditions to highlight either an intrinsic cause (e.g., “Look, the Yellows got a point! Yellows are good at throwing, not Purples”), an extrinsic cause (e.g., “Look, the Yellows got a point! This game has high-up hoops, not low-down ones!”) or neither cause (e.g., “Look, the Yellows got a point!”). The experimenter provided the framing three times, after each point scored.

#### Test Phase

Finally, participants were asked a series of five questions in a fixed order: a manipulation check, two attribution questions (explanation and intervention), and two social attitude questions (friendship preference and prize allocation). First, participants were asked to recall the outcome (“Who won the game?”) and responded verbally or by pointing, ensuring that they correctly recalled the status disparity. Next, participants were asked to freely explain the outcome (“Why did they win the game?”). Participants who simply restated the victory (e.g., “Because they had more points”) were re-prompted (e.g., “Why did they have more points?”). Participants were then asked for an intervention that would result in a different outcome. We avoided asking participants what they could change to alter the outcome, as we reasoned that this might capture participants’ beliefs about whether intrinsic or extrinsic causes were more malleable. Instead, we asked participants, what could *be different* that would result in a different outcome (“What could be different so that the other kind could win?”). Finally, participants were asked whom they preferred as a friend (“Who do you want to be friends with?”) and to whom they would award a prize (“Who would you give a prize to?”).

The test phase concluded a trial. Following Trial 1, participants completed Trials 2 and 3. All trials followed the same procedure but involved different materials, featuring a different set of characters and a different set of structures: Trial 2 featured the Blues/Oranges (with speedy hands/feet making them good at climbing/running) playing a capture-the-flag game (with vertical/horizontal flagpoles requiring climbing/running). Trial 3 featured the Reds/Greens (who were tall/short making them good at jumping over/sliding under), running a race (with high/low obstacles requiring jumping/sliding). So, all trials featured a victory that could be either be attributed to an intrinsic or an extrinsic cause.

### Coding

Responses to attribution questions were transcribed by Research Assistants and coded by the first author as personal, structural, or combinational, with the condition concealed. Responses referring to characters – their bodies, abilities, or behaviors – were coded as personal, and responses referring to the game – its physical components, rules, or required behaviors – were coded as structural. See Table 1 for examples of coded responses from Trial 1. Overall, 58% of responses were coded as personal and 36% were coded as structural. During data collection, but prior to data analyses, we noticed that a small number of responses referred to both causes (6% of responses), and we decided to code these as combinational. To determine reliability, a subset of responses (one-third) was coded by the second author, also with the condition concealed. The two raters agreed on 93% of codes, yielding a Cohen’s kappa of 0.86.

**Table 1:** Examples of responses coded as personal, combinational, and structural.

	Explanations (Responses to “Why did they win the game?”)	Interventions (Responses to “What could be different so that the other kind could win?”)
Coded as Personal	“Because they are stronger at throwing.” “Because they have strong muscles.” “Better at throwing.” “Because they throw.” “They have arms.” “Cause they can throw really good.” “They got so many shots because of their hands.”	“Switch the arms and legs.” “Their legs could be strong and their arms could be strong.” “We would need to change the feet.” “Change the Purples’ hands and feet.” “Make them kick really hard.” “If they were good at throwing.” “If the Yellows had stronger legs and the Purples had stronger arms.” “Make the Purples throw better than the Yellows.”
Coded as Combinational	“Because you throw not kick, and the baskets are high up.” “Cause they were good at throwing cause the hoops were high up, but the Purple cannot throw.” “Because it was a throwing game and they are really good at throwing.”	“They were not really good at anything. We could give them lower down ones so it’d be a little bit easier for them.” “They have to kick so put the hoops on the ground.”
Coded as Structural	“Because there was only high baskets.” “They were all high.” “Because there were no low- down baskets for them to kick it in.” “That one had throwing hoops.” “Up-tall poles.” “Hoops are for throwing.” “The scoregoal thingy was high-up.”	“Make the goal be under.” “Make it a kicking game.” “The rings would have to be down.” “Well, the way I’d make it so maybe one wins or maybe the other wins, but there’s be a low- down hoop and a high-up hoop on each side.” “If the hoops were downer.” “Switch sides.” “Put the hoops on the ground.”

## Results

Analyses focused on how verbal framing condition impacted attributions, and in turn, social attitudes. By including age and trial number as predictors in our models, we also explored our secondary research questions concerning effects of age and participants’ ability to generalize the verbal framing they received to the third (unframed) trial. All analyses were conducted in Jamovi, an open-source R-based statistical analysis package (The Jamovi Project, 2020).

### Impact of Framing Condition on Attributions

To assess the impact of framing condition on participants’ attributions, we fit a logistic mixed-effects model with condition (Intrinsic vs. Neutral and Extrinsic vs. Neutral), age (continuous), attribution question (Explanation vs. Intervention), trial number (1 vs. 2 and 1 vs. 3), the interaction between condition and age, and the interaction between condition and trial number as fixed effects. The model also included random intercepts for participants. Attributions were coded either personal (“1”) or structural (“0”). Combinational responses were excluded from this model, following the approach used in prior work (Peretz-Lange and Muentener, 2019).

**Figure 3:**
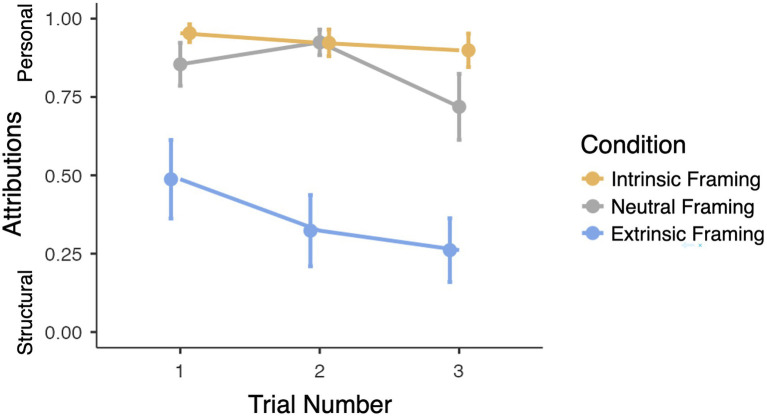
Impact of framing condition and trial number on attributions. Higher scores reflect more personal attributions, and lower scores reflect more structural attributions. Error bars represent +/− 1 SE.

The model revealed significant main effects of framing condition [*X*^2^(2)=24.33, *p*<0.001], age [*X*^2^(1)=10.1, *p*=0.001], attribution question [*X*^2^(1)=82.69, *p*<0.001], and trial number [*X*^2^(2)=8.66, *p*=0.013]. The impact of framing condition was asymmetrical such that participants provided significantly more structural attributions in the Extrinsic condition (58.7%) compared to the Neutral condition [31%; *b*=−2.34, *SE*=0.63, 95% CI (−3.57, −1.12), *p*<0.001], but these rates did not differ between the Intrinsic condition (24.4%) and Neutral conditions (31%; *p*=0.19), in line with our hypothesis. Increased age was associated with increased structural and reduced personal attributions [*b*=−1.52, *SE*=0.48, 95% CI (−2.47, −0.59), *p*=0.001]. Attribution question type predicted significant variance in responses, with intervention questions more likely to yield structural responses (55.3% of responses) compared to explanation questions [18.9%, *b*=−3.05, *SE*=0.34, 95% CI (−3.71, −2.39), *p*<0.001]. Finally, although attributions did not differ between the first and second trials (34.9% compared to 35.5%, *p*=0.60), participants were significantly more likely to provide structural explanations on the third trial (43.4%) compared to the first trial [34.9%, *b*=−0.89, *SE*=0.32, 95% CI (−1.51, −0.26), *p*=0.01]. Notably, however, framing condition did not significantly interact with trial type (*p*=0.29), indicating that framing continued to impact attributions on the third unframed trial, in line with our prediction. The interaction between age and framing condition was also not significant (*p*=0.48). Overall, as shown in Figure 3, participants provided more structural attributions in the Extrinsic framing condition compared to the Neutral and Intrinsic framing conditions, across all trials.

Although this model excluded combinational responses, a secondary model was conducted utilizing a wider coding scheme (1=personal and 0=structural *or combinational*), considering any response mentioning structural causes as structural, which enabled us to make use of all available data and to confirm the robustness of the results. This secondary model revealed many of the same fixed and random effects as the primary model, including significant main effects of framing condition [*X*^2^(2)=22.24, *p*<0.001], age [*X*^2^(1)=12.27, *p*<0.001], and attribution question [*X*^2^(1)=66.46, *p*<0.001]. The impact of framing condition was asymmetrical such that participants provided significantly more structural attributions in the Extrinsic (60.9%) compared to the Neutral condition [35.2%, *b*=−1.76, *SE*=0.50, 95% CI (−2.67, −0.78), *p*<0.001], but these rates did not differ between the Intrinsic (28%) and Neutral conditions (35.2%, *p*=0.23), in line with our hypothesis. Increased age was associated with increased structural and reduced personal attributions [*b*=−1.34, *SE*=0.38, 95% CI (−2.09, −0.59), *p*<0.001]. The type of attribution question predicted significant variance in responses, with intervention questions more likely to yield structural responses (55.8% of responses) than explanation questions [26.9% of responses, *b*=−2.10, *SE*=0.26, 95% CI (−2.61, −1.59), *p*<0.001]. Finally, there were no significant interactions between framing condition and trial type (*p*=0.08) or between framing condition and age (*p*=0.72). In one deviation from the results of the primary model, trial number did not reach significance in this model (*p*=0.08). Thus, the effects revealed in the primary model were robust across stricter and broader approaches to coding structural responses that either excluded or included combinational responses.

As we were interested in the conceptual depth of our manipulation on participants’ attributions, we conducted exploratory content analyses of participants’ explanations to determine the proportion that were direct imitations of the provided verbal framing. We coded direct imitations in a sample of 70 explanations, the explanations from Trial 1 in just the Intrinsic and Extrinsic framing conditions, as there was no verbal framing to imitate in the Neutral framing condition (we also did not code interventions, as directly imitating the verbal framing would not be a suitable response to the intervention question). To qualify as direct imitations, responses needed to repeat the experimenter’s language – in the intrinsic framing condition, the phrase “good at throwing,” and in the extrinsic framing condition: the phrase “high-up hoops” – and contain no additional meaningful novel content. Only eight out of the 70 explanations were coded as direct imitations. Thus, participants’ explanations usually included novel content, and only rarely represented direct imitations of the experimenter’s verbal framing. These results support the validity of participants’ responses as a meaningful reflection of their reasoning, in line with other research using children’s explanations as a window into their causal reasoning.

### Impact of Framing Condition on Social Attitudes

Next, we evaluated our hypothesis that framing condition would impact social attitudes. To do so, we fit a logistic mixed-effects model with framing condition (Intrinsic vs. Neutral and Extrinsic vs. Neutral), age (continuous), attitude question type (Friendship Preference vs. Prize Allocation), and trial number (1 vs. 2 and 1 vs. 3) as fixed effects. The model also included random intercepts for participants. Responses to social attitude questions were coded either choosing the dominant group (“1”) or the subordinate group (“0”). The model revealed a significant main effect of attitude question type [*X*^2^(1)=8.44, *p*=0.004], with participants more likely to give a prize to the dominant group (63.2% of trials) than to befriend a dominant group member (52.4% of trials) but no other significant effects. Reliability analyses also revealed that internal reliability among these two questions was low (Cronbach’s alpha=0.14), in contrast to our expectation that both measures would capture social attitudes.

**Figure 4:**
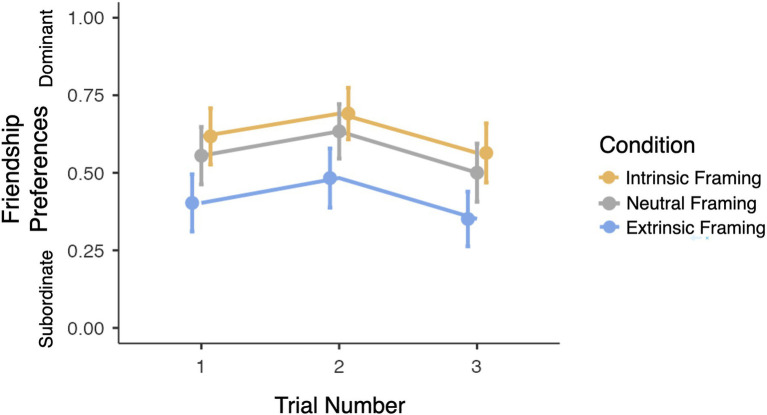
Impact of framing condition on friendship preferences. Higher scores represent preferences moreso favoring the dominant over the subordinate group. Scores of 0.5 represent chance performance on the forced-choice trials. Error bars represent +/− 1 SE.

Following this result, two exploratory mixed-effects models were fit to participants’ friendship preferences and prize allocations separately. As shown in Figure 4, the model of participants’ friendship preferences revealed a significant main effect of framing condition [*X*^2^(2)=6.65, *p*=0.036]. In particular, participants in the Extrinsic framing condition were significantly less likely to befriend a dominant group member (as they did on 43.8% of trials) compared to participants in the Neutral framing condition [as they did on 54.6% of trials, *b*=−12.03, *SE*=5.28, 95% CI (−22.39, −1.68), *p*=0.02], but no such difference was revealed between participants in the Intrinsic and Neutral framing conditions (58.8 vs. 54.6%, *p*=0.86). This effect of framing condition was qualified by a marginally significant interaction between framing condition and age [*X^2^*(2)=5.93, *p*=0.051]; specifically, the effect of the Extrinsic framing condition relative to the Neutral framing condition grew significantly less strong over development [*b*=1.92, *SE*=0.88, 95% CI (0.19, 3.66), *p*=0.03], but no such age-related change was observed in the effect of the Intrinsic framing condition relative to the Neutral framing condition (*p*=0.82). However, this interaction did not reach significance and should be interpreted with caution. No other significant main effects or interactions were revealed. The model of participants’ prize allocations revealed no significant main effects or interactions.

## Discussion

The present study investigated how the verbal framing of novel social status disparities impacted children’s personal vs. structural attributions for the disparities, and in turn, their social attitudes toward novel dominant and subordinate groups. Results indicated that verbal framing significantly and asymmetrically impacted participants’ attributions for the disparities: Participants formed mostly personal attributions in the neutral and intrinsic framing conditions, but relatively more structural attributions in the extrinsic framing condition. Participants also generalized the verbal framing they received to a new unframed scenario, and attributions grew more structural over development, both in line with our predictions. Results also showed that framing did not impact participants’ social attitudes in general; however, framing did impact participants’ friendship preferences in particular. We discuss each set of results, below.

### Impact of Framing on Attributions

Supporting our first primary hypothesis, results indicated that verbal framing condition had a significant and asymmetrical impact on participants’ attributions for the novel status disparities. Participants tended to make mostly personal attributions in the Neutral and Intrinsic framing conditions, no different from one another and in line with their default essentialist intuitions but made significantly more structural attributions in the Extrinsic framing condition. Analyses also revealed age-related increases in structural attributions.

Prior work on children’s structural attributions has primarily involved teaching children explicitly that disparities that were caused by either intrinsic or extrinsic factors (e.g., Hussak and Cimpian, 2015; Sutherland and Cimpian, 2019; Rizzo et al., 2020; Dunlea and Heiphetz, 2021). However, recent reviews have pointed out that children rarely receive explicit information about causes of disparities (Elenbaas et al., 2020). Instead, children may encounter disparities with ambiguous causal origins and are left to their own devices to form attributions for them. We simulated this encounter in the present study by introducing children to status disparities that could rationally be attributed to either an intrinsic or an extrinsic cause. We found that in the face of this ambiguous evidence, subtle verbal framing robustly shaped children’s attributions for the disparities.

This finding adds to a growing body of work demonstrating that extrinsic verbal framing can be a powerful tool for disrupting children’s essentialist intuitions about a variety of intergroup differences (e.g., intergroup differences in behavior, Vasilyeva et al., 2018). Here, we demonstrate its impacts on children’s reasoning about intergroup differences in status in particular. In doing so, we leveraged research on children’s causal reasoning showing that children spontaneously form causal explanatory theories to account for observed phenomena (Schulz and Sommerville, 2006; Muentener and Schulz, 2014) but that verbal framing can promote new causal-explanatory theories that displace children’s default theories (Butler and Markman, 2012; Garvin and Woodward, 2015; Peretz-Lange and Muentener, 2019).

### Impacts of Framing on Social Attitudes

We did not find evidence that framing significantly impacted social attitudes directly, when both measures of social attitudes were included in our model. One possible interpretation of these results is that perhaps framing exerted only a superficial impact on participants’ attributions, and this is why it did not have downstream consequences on social attitudes. We do not believe this is the case, for several reasons: First, verbal framing impacted not only participants’ explanations (which could have taken the form of direct imitations of the framing) but also their interventions (which required producing novel content). Second, rates of direct imitations, even within participants’ explanations, were low. Finally, the impact of verbal framing extended to a third unframed trial, suggesting that it shaped participants’ conceptual understanding of the kinds of factors shaping the disparities, rather than merely drawing their perceptual attention to different aspects of the visual scene. For these reasons, we believe that framing exerted a conceptually deep impact on participants’ attributional reasoning.

Instead, we interpret this result as reflecting a weakness in our social attitude measures. Recall that we used two social attitude measures, a friendship preference measure and a prize allocation measure. This design choice was based on prior work finding strong internal reliability among questions about children’s liking of a novel high-status group and their belief that the group deserved its advantage (Hussak and Cimpian, 2015, Study 4), as well as work showing that social essentialism may have particularly strong impacts on resource-sharing (Rhodes et al., 2018). The prize allocation measure used in this study was designed as a thematic adaptation of a resource-sharing measure, with a prize being a resource that is often given to a high-achieving or dominant group. However, the prize allocation measure may not have functioned as intended. Internal reliability between friendship preferences and prize allocations was low, in contrast to our intention for both measures to capture the construct of social attitudes. One *post-hoc* interpretation of this result is that the awarding of prizes does not really reflect liking (as in other examples of resource-sharing), but rather reflects the fact of having won a game. Perhaps the convention of awarding prizes to winners may have been too powerful for children to overcome.

Exploratory analyses revealed that framing condition did significantly impact friendship preferences but did not significantly impact prize allocations. Impacts on friendship preferences followed the predicted asymmetrical pattern. We believe that this result, taken in the context of the prior results, provides tentative exploratory support for the possibility that verbal framing of status disparities may impact children’s social attitudes. However, this *post-hoc* interpretation should be received with caution, given that our *a priori* hypothesis was that framing would impact social attitudes overall, across both measures. Future research is needed to better understand how attributions may differentially shape different aspects of social attitudes.

### Limitations

Several important limitations are of note. First, attributions represent only one component of essentialist reasoning. Essentialist reasoning involves a host of cognitive processes beyond attributions to intrinsic causes (for discussions, see Quillien, 2018; Noyes and Keil, 2020), such as beliefs in within-group homogeneity, sharp group boundaries, heritability of group membership, stability of group membership, and more. We focused on the intrinsic attributional bias here, as it may play a central role in essentialist theories (Cimpian and Salomon, 2014; Sutherland and Cimpian, 2019), and it provides an opportunity to displace children’s default attributions with alternative attributions. However, we do not claim that this manipulation mitigated social essentialist reasoning in general, but merely that it impacted children’s attributions.

Along the same lines, although the present study contrasts essentialist with structural reasoning in terms of the attributions children form, we note that structural reasoning is not an antidote to all components of social essentialist reasoning. In fact, some work has found that structural reasoning can function similarly to essentialist reasoning, in that it can support judgments of within-group similarity, normative social kind representations, and stereotyping (see Yzerbyt et al., 1998; Haslanger, 2015; Vasilyeva et al., 2018 for discussions). Future research should continue to explore the social consequences of structural attributions.

Next, we note that although our results indicated that children tended to provide personal attributions for the disparities presented, this might partially reflect the nature of our sample, which consisted of mostly White children from middle- and upper-class backgrounds. Some researchers view essentialism as a motivated worldview that reflects people’s desire to view their ingroup positively (Keller, 2005) or justify the social order (Yzerbyt et al., 1997; Williams and Eberhardt, 2008). Thus, it is conceivable that children who are from high-status backgrounds themselves, as many of our participants were, might have particularly strong essentialist tendencies, which presented themselves even in a novel groups context. We are not aware of research finding racial or SES-related differences in children’s essentialism (in fact, see Pauker et al., 2016 and Dunlea and Heiphetz, 2021 for evidence to the contrary), but future research should nevertheless employ a diverse sample to confirm that children’s own social status did not affect their attributional reasoning about status.

Next, although our novel groups paradigm was designed to avoid children’s prior knowledge of familiar disparities and determine their attributions when both intrinsic and extrinsic causes were plausible, this novel groups approach was also limited in several important respects. First, we used winning/losing to represent status (specifically, social dominance) in this novel paradigm, reasoning that this would clearly connote a dominance hierarchy to children even in a completely novel context. This approach follows prior developmental work operationalizing novel status hierarchies as winning/losing (e.g., Thomas et al., 2018; Thomsen, 2020). Children, infants, and even nonhuman animals use winning/losing to infer a group’s status and position in a social hierarchy (see Thomsen, 2020 for a review), suggesting that this operationalization taps into foundational conceptions of social status. However, this approach is also limited in its direct applicability to real social status disparities, such as race- and gender-related disparities, which are intimately related social power (Gülgöz and Gelman, 2017) and wealth (Shutts et al., 2016), not just dominance. Although children’s conceptions of status are similar across these different dimensions (Enright et al., 2020), future research should explore children’s reasoning about these other dimensions of social status as well.

Second, in contrast to our novel status disparities, real-world social disparities are *not* equally attributable to either groups’ intrinsic deficiencies or their extrinsic obstacles. They also are not arbitrary, as in the present study, but rather reflect the systematic marginalization of low-status groups by high-status groups, for their own gain. Real-world structural causes are not concrete objects but are instead abstract norms, cultures, and sometimes-subtle behaviors that have impacts only cumulatively. These important features of real-world status disparities were not present in the novel groups paradigm used in the present study. The novel groups approach was not designed to be a perfect analogy for real status disparities but was instead designed to reveal children’s attributional reasoning in a context in which neither cause was better at accounting for the observed disparity, in which neither cause was more salient, and in which children had no previous exposure to any messages about the disparity or its cause. The fact that participants showed a strong tendency toward personal attributions *even* in this context demonstrates the robustness of their personal attributional tendencies. On the other hand, the fact that extrinsic verbal framing mitigated this attributional tendency in the present study is optimistic; all the moreso should teaching children about extrinsic causes mitigate children’s essentialist reasoning in the real social world, where extrinsic causes actually do account for disparities better than intrinsic causes. In sum, although the present novel groups approach sacrifices some external validity, it allowed us to carefully characterize children’s attributional reasoning.

## Conclusion

Despite the vastness of structural inequalities in society, research on children’s structural reasoning is slim. The present study provides initial evidence that, when encountering status disparities of uncertain causal origins, children tend to form personal attributions for them, and that these attributions are related to prejudiced social attitudes. Optimistically, though, we demonstrate that extrinsic framing may be a powerful tool in disrupting this default process and enabling children to view low-status groups as disadvantaged rather than inferior.

## Data Availability Statement

Datasets presented in this article are not publicly available because of IRB restrictions on public sharing of data from child participants. Requests to access the datasets should be directed to the first author at Rebecca.PeretzLange@Purchase.edu.

## Ethics Statement

The studies involving human participants were reviewed and approved by the Tufts University Social, Behavioral, and Educational Research (SBER) IRB, Protocol #1801006. Written informed consent to participate in this study was provided by the participants’ legal guardian/next of kin.

## Author Contributions

RP-L designed the study, with feedback from PM. RP-L collected data, analyzed the results, and wrote the first draft of the manuscript. PM contributed to manuscript revision, read, and approved the submitted version. RP-L submitted the manuscript. All authors contributed to the article and approved the submitted version.

## Funding

The publication of this research was supported by an Open Access Publishing Award from the Faculty Research Awards Committee at Tufts University.

## Conflict of Interest

The authors declare that the research was conducted in the absence of any commercial or financial relationships that could be construed as a potential conflict of interest.

## Publisher’s Note

All claims expressed in this article are solely those of the authors and do not necessarily represent those of their affiliated organizations, or those of the publisher, the editors and the reviewers. Any product that may be evaluated in this article, or claim that may be made by its manufacturer, is not guaranteed or endorsed by the publisher.
